# *Trigonocranus emmeae* Fieber, 1876 (Hemiptera, Fulgoromorpha, Cixiidae) – a new species for Poland

**DOI:** 10.3897/zookeys.319.4278

**Published:** 2013-07-30

**Authors:** Krzysztof Musik, Marcin Walczak, Łukasz Depa, Łukasz Junkiert, Anna Jedynowicz

**Affiliations:** 1 Department of Zoology, Faculty of Biology and Environmental Protection, University of Silesia, Bankowa 9, 40-007 Katowice, Poland; 2 Brzezinka, ul. Skotnicka 3A 32-600 Oświęcim, Poland

**Keywords:** Faunistics, new records, *Trigonocranus*, Cixiidae, Fulgoromorpha

## Abstract

Single macropterous female of *Trigonocranus emmeae* Fieber, 1876 has been found during the faunistic studies in semi-natural plant communities of Oświęcim city in southern Poland. It is the first record of this species in Poland. *Trigonocranus emmeae* is rarely collected within the wide range of its distribution, mostly due to its hidden life mode.

## Introduction

The Fulgoromorpha and Cicadomorpha are represented in Europe by 2053 species (Hoch 2012). In Poland the fauna of Auchenorrhyncha comprises 537 recorded species, including the latest checklist by Chudzicka (2004) and species that were missed (Łabanowski and Soika 1997, Gębicki 2003, Świerczewski and Gebicki 2003) or published later than 2004 (Gaj et al. 2009, Świerczewski and Stroiński 2011a, 2011b, Świerczewski and Walczak 2011, Walczak et al. 2012).

During the recent studies in Brzezinka, a suburban district of Oświecim city (southern Poland) a single female of *Trigonocranus emmeae* Fieber, 1876 has been collected. It is the first record of this poorly studied representative of Cixiidae in Poland.

## Material and methods

Faunistic studies on planthoppers and leafhoppers (Cicadomorpha and Fulgoromorpha) were conducted during the vegetation season of 2008. The area of the research was a suburban district of Oświęcim city – Brzezinka (UTM:CA64) (50°2'51"N, 19°9'38"E).

Insects were collected by a standard sweeping net (Ø 35cm) from the end of April till the end of October, altogether 15 samples per plot were taken. The collected material was transferred to a container with ethyl acetate. In the laboratory the collected insects were mounted on glue boards and determined. The key used to identify the species was Biedermann and Niedringhaus (2004).

Chorological and ecological data used in this work are accordant to Nickel and Remane (2002) and Nickel (2003). To determine the plant associations the key provided by Matuszkiewicz (2008) was applied.

Collected material is deposited in the Collection of Department of Zoology, University of Silesia, Katowice.

## Results

**Figure 1.**
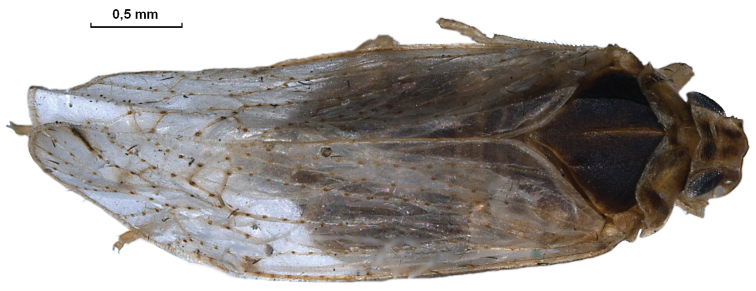
*Trigonocranus emmeae* macropterous female (photo by A. Stroiński).

*Trigonocranus emmeae* Fieber, 1876 – new species for Polish fauna (Fig. 1).

## Material examined

A single specimen of this species was collected on 22.06.2008 in Oświęcim-Brzezinka (southern Poland), [UTM CA64], 50°2'51"N, 19°9'38"E, in the meadow belonging to the *Molinio-Arrhenatheretea* class, macropterous ♀, leg. A. Jedynowicz, det. M. Walczak, C. Gębicki rev. (specimen deposited in the collection of Department of Zoology, University of Silesia).

**Figure 2.**
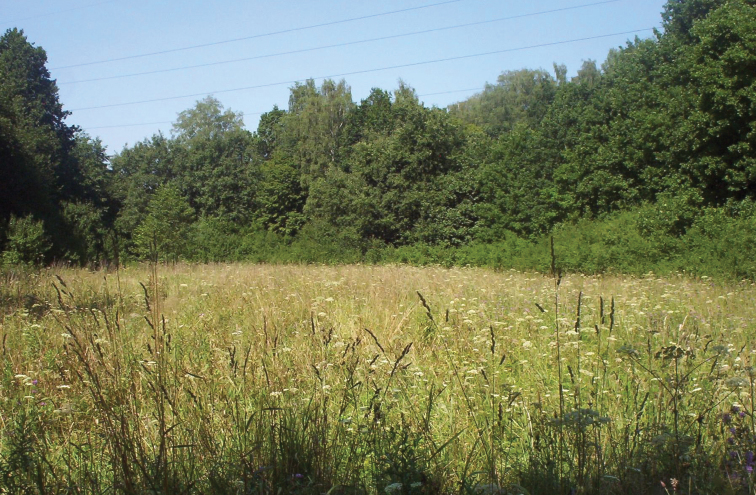
Habitat of *Trigonocranus emmeae*, Oświęcim-Brzezinka (photo by A. Jedynowicz).

The first locality of *Trigonocranus emmeae* in Poland was located in Oświęcim-Brzezinka, Leśna street (Fig. 2). It was a *Molinio-Arrhenatheretea* class meadow surrounded by forest. The dominant plant species were: *Holcus mollis*, *Dactylis glomerata*, *Alopecurus pratensis* and *Agrostis capillaris*, in less percentage: *Anthoxanthum odoratum*, *Elymus repens*, *Carex hirta*, *Carex acutiformis* and *Carex vulpia*. During the studies in 2008 apart from *Trigonocranus emmeae* there were 36 other species recorded in this plot. The dominant species were: *Cicadella viridis* (Linnaeus, 1758) (23.57% of collected material), *Cicadula quadrinotata* (Fabricius, 1794) (8.02%), *Arthaldeus pascuellus* (Fallén, 1826) (5.02%) and *Stenocranus major* (Kirschbaum, 1868) (3.31%) (Jedynowicz 2009).

## Discussion

According to the literature *Trigonocranus emmeae* is widely distributed in Europe, but very rarely collected. This species was recorded in Austria, Switzerland, France, Great Britain (Nast 1972), Slovenia (Holzinger and Seljak 2001, Seljak et al. 2003), Croatia, Spain, Italy, Bulgaria, Germany, Georgia, southern Russia (Holzinger et al. 2003, Nickel 2003), Czech Republic (Malenovský and Lauterer 2010, 2012), Luxemburg (Niedringhaus et al. 2010) and Sweden (Larsson 2010).

According to Nickel (2003) nymphs and brachypterous adults are unpigmented with reduced number of ommatidia and live on the soil surface and leaf litter. This species occurs from the end of May till the end of July, hibernates in the egg stage and is univoltine. *Trigonocranus emmeae* represents the European chorological element. It lives on moderately warm sites covered by vegetation of medium density, probably feeding on roots of shrubs. The mentioned specimen was collected on a moderately moist meadow. The data from Great Britain indicates that this species may be also collected in damp sites (Bantock 2012). The host plant species is unknown (Nickel and Remane 2002, Nickel 2003). There are no data about the trophic relations of this species, therefore it is unknown if it is mono-, oligo- or most probably polyphagous. The majority of collected specimens were caught by sweeping net or Malaise traps during the dispersal flight (Nickel 2003). *Trigonocranus emmeae* can be also and effectively collected by an underground pitfall trap (M. Wilson, pers. comm.).

The difficulties in collecting *Trigonocranus emmeae* result in lack of detailed biological and ecological data. This species is probably not as rare as it seems, however the right collection method must be applied to reveal its cryptic presence. Distribution of this species in Poland needs further research.
